# The Association of Dietary l-Arginine Intake and Serum Nitric Oxide Metabolites in Adults: A Population-Based Study

**DOI:** 10.3390/nu8050311

**Published:** 2016-05-20

**Authors:** Parvin Mirmiran, Zahra Bahadoran, Asghar Ghasemi, Fereidoun Azizi

**Affiliations:** 1 Nutrition and Endocrine Research Center, Research Institute for Endocrine Sciences, Shahid Beheshti University of Medical Sciences, Tehran 19395-4763, Iran; mirmiran@endocrine.ac.ir; 2 Endocrine Physiology Research Center, Research Institute for Endocrine Sciences, Shahid Beheshti University of Medical Sciences, Tehran 19395-4763, Iran; ghasemi@endocrine.ac.ir; 3 Endocrine Research Center, Research Institute for Endocrine Sciences, Shahid Beheshti University of Medical Sciences, Tehran 19395-4763, Iran

**Keywords:** l-arginine, nitric oxide, nitrate, nitrite

## Abstract

This study was conducted to investigate whether regular dietary intake of l-arginine is associated with serum nitrate + nitrite (NOx). In this cross-sectional study, 2771 men and women, who had participated in the third examination of the Tehran Lipid and Glucose Study (2006–2008), were recruited. Demographics, anthropometrics and biochemical variables were evaluated. Dietary data were collected using a validated 168-food item semi-quantitative food frequency questionnaire and dietary intake of l-arginine was calculated. To determine any association between dietary l-arginine and serum NOx, linear regression models with adjustment for potential confounders were used. Mean age of participants (39.2% men) was 45.9 ± 15.9 years. After adjustment for all potential confounding variables, a significant positive association was observed between l-arginine intake and serum NOx concentrations in the fourth quartile of l-arginine (β = 6.63, 95% CI = 4.14, 9.12, *p* for trend = 0.001), an association stronger in women. Further analysis, stratified by age, body mass index and hypertension status categories, showed a greater association in middle-aged and older adults (β = 9.12, 95% CI = 3.99, 13.6 and β = 12.1, 95% CI = 6.48, 17.7, respectively). l-arginine intakes were also strongly associated with serum NOx levels in overweight and obese subjects in the upper quartile (β = 10.7, 95% CI = 5.43, 16.0 and β = 11.0, 95% CI = 4.29, 17.5); a greater association was also observed between l-arginine intakes and serum NOx in non-hypertensive (HTN) compared to HTN subjects (β = 2.65, 95% CI = 2.1–3.2 *vs.* β = 1.25, 95% CI = −1.64–4.15). Dietary l-arginine intakes were associated to serum NOx and this association may be affected by sex, age, body mass index, and hypertension status.

## 1. Introduction

l-arginine is a conditionally essential amino acid and its deficiency has been reported to be related to a variety of inflammatory and oxidative processes leading to metabolic disorders and cardiovascular diseases [1,2,3]. Besides its availability from dietary intakes, l-arginine is provided from endogenous pathways, including protein turnover and *de novo* synthesis from citrulline in the urea cycle [4]. l-Arginine is involved in production of nitric oxide (NO), an important signaling molecule in biological pathways, as the substrate of NO synthase (NOS) enzymes family [5,6]. l-arginine/NO pathway is involved in many physiological processes and any changes in this pathway can be related to development of diseases [7,8,9,10,11]. In our previous studies, we showed that altered NO metabolism, characterized as increased serum NOx levels, may be related to some pathological conditions [12,13].

Basal NO synthesis rate in healthy subjects has been estimated to be in the range of 13–65 μmol/h; plasma l-arginine supplies 54% of the substrate used for whole daily NO synthesis [14], and contribution of dietary l-arginine intake in the daily basal NO synthesis was also reported at approximately 2.7%–5.7% (1.1–2.3 μmol/h during the 8 h following a low-and high-arginine diet, respectively) [15,16]. Direct utilization of dietary l-arginine in NO synthesis seems to be low, and reduced l-arginine intake may however limit NO production due to a large dependence of intracellular l-arginine pool to extracellular sources [14,17,18].

The association of l-arginine intakes and systemic NO production is currently an important gap of knowledge with controversial data. Modulation of NO-related pathways following oral l-arginine supplementation has led to the current dominant belief that oral arginine intake dose-dependently modulates arginine bioavailability and promotes NO synthesis [19,20]; on the other hand, this relation is doubtful because NOS enzyme should theoretically be saturated in the presence of physiological concentrations of l-arginine [21]; findings from previous studies, investigating the effects of l-arginine supplementation on improvement of NO production, are inconsistent and could not yet respond to this challenging paradigm, currently known as the “l-arginine paradox” [16,22,23]. To the best of our knowledge, this issue has not yet been investigated in the framework of a population-based study, the results of which could provide useful information regarding the association between dietary intake of l-arginine and NO synthesis. Hence, the main focus in this study was to assess whether usual intake of l-arginine is associated with serum nitrate + nitrite (NOx) concentration, an indicator of systemic NO production, in a population of Iranian adults.

## 2. Methods

### 2.1. Study Population

This study was conducted within the framework of the Tehran Lipid and Glucose Study (TLGS), an ongoing community-based prospective study being conducted to investigate and prevent non-communicable diseases, in a representative sample in district 13 of Tehran, the capital city of Iran [24]. In the current study, adult participants (≥20 years) of the third (2006–2008) TLGS examination, with measurements of serum NOx and completed data on dietary intakes were enrolled. We excluded pregnant women and subjects who had chronic or frequent diarrhea. Participants were also excluded from the final analysis if they reported implausible energy intake (<800 kcal/day or ≥4200 kcal/day) or were on specific diets or consumed any dietary supplements. Final analysis was conducted on 2771 subjects (1086 men and 1685 women).

### 2.2. Ethics, Consent and Permissions

Written informed consents were obtained from all participants and the study protocol was approved (ethics committee number: 57ECRIES94/02/15) by the ethics research council of the Research Institute for Endocrine Sciences, Shahid Beheshti University of Medical Sciences.

### 2.3. Demographic and Anthropometric Measures

Trained interviewers collected demographic and medical history information from participants using pretested questionnaires. Smoking status was obtained during face-to-face interviews; subjects who smoked daily or occasionally were considered current smokers, while non-smokers included those who had never smoked or those who had quit smoking. Information on medication usage for treatment of thyroid disorders, diabetes, hypertension (HTN) and lipid disorders was also collected.

Anthropometric measurements were assessed by trained staff. Weight was measured to the nearest 100 g using digital scales while the subjects were minimally clothed, without shoes. Height was measured to the nearest 0.5 cm in a standing position without shoes using a tape meter. Body mass index (BMI) was calculated as weight (kg) divided by square of the height (m^2^). Waist circumference was measured to the nearest 0.1 cm, midway between the lower border of the ribs and the iliac crest at the widest portion, over light clothing, using a soft measuring tape, without any pressure to the body. For blood pressure (BP) measurements, after a 15-min rest in the sitting position, two measurements of BP were taken, on the right arm, using a standardized mercury sphygmomanometer; the mean of the two measurements was considered as the participant’s BP.

### 2.4. Biochemical Measures

Fasting blood samples were taken after 12–14 h from all study participants at baseline and follow-up phases. Fasting serum glucose was measured by the enzymatic colorimetric method using glucose oxidase. The standard 2 h serum glucose test was performed for all individuals who were not on anti-diabetic drugs. Serum total cholesterol (TC) and triglycerides level (TG) were measured by enzymatic colorimetric analysis with cholesterol esterase and cholesterol oxidase and glycerol phosphate oxidase, respectively. High-density lipoprotein cholesterol (HDL-C) was measured after precipitation of the apolipoprotein B containing lipoproteins with phosphotungstic acid. Low-density lipoprotein cholesterol (LDL-C) was calculated from serum TC, TG and HDL-C, according to the Friedewald equation. Analyses were performed using Pars Azmoon kits (Pars Azmoon Inc., Tehran, Iran) and a Selectra 2 auto-analyzer (Vital Scientific, Spankeren, The Netherlands). Both inter- and Intra-assay coefficients of variations of the assays were <5%.

Serum creatinine levels were assayed using kinetic colorimetric Jaffe method. Serum NOx concentration was measured by a rapid, simple spectrophotometric method developed by Miranda *et al.* [25], and had been validated in our laboratory [26,27]. Inter- and Intra-assay coefficients of variations of the assays were 5.2% and 4.4%, respectively. The sensitivity of the assay was 2.0 μmol/L and its recovery was 93% ± 1.5% [28].

### 2.5. Dietary Assessment

A validated 168-item food frequency questionnaire (FFQ) was used to assess typical food intakes over the previous year. Trained dietitians, with at least five years of experience in the TLGS survey, asked participants to designate their intake frequency for each food item consumed during the past year on a daily, weekly, or monthly basis. Portion sizes of consumed foods reported in household measures were then converted to grams [29]. The validity of the food frequency questionnaire has previously been evaluated by comparing food groups and nutrient values determined from the questionnaire with values estimated from the average of twelve 24-h dietary recall surveys and the reliability has been assessed by comparing energy and nutrient intakes from two FFQ; Pearson correlation coefficients and intra-class correlation for energy and nutrients showed acceptable agreements between FFQ and twelve 24-h dietary recall surveys, and FFQ1 and FFQ2 [30]. However, since Iranian Food Composition Table is incomplete, and has limited data on nutrient content of raw foods and beverages, to analyze foods and beverages for their energy and nutrient content, we used the US Department of Agriculture Food Composition Table [31].

Dietary intakes of main nitrate- and nitrite-containing foods such as vegetables, grains and processed meats were determined and considered as confounding variables. Nitrate containing vegetables (NCVs) as well as its categories including low-nitrate (<50 mg/100 g fresh weight of vegetables), medium-nitrate (50–100 mg/100 g fresh weight of vegetables) and high-nitrate (>100 mg/100 g fresh weight of vegetables) were calculated [32]. Low-nitrate vegetables included potatoes, broad beans, tomato, ketchup, cucumber, squash, eggplant, string beans, carrots, garlic, onions, pepper, mushroom and watermelon; medium-nitrate vegetables included cabbage and turnip; and high-nitrate vegetables include celery, lettuce, and spinach.

### 2.6. Definition of Terms

Overweight and obesity were defined as 25 ≤ BMI < 30, and BMI ≥ 30 kg/m^2^, respectively. Diabetes was defined as fasting serum glucose ≥126 mg/dL, 2 h serum glucose ≥200 mg/dL or anti-diabetic medications [33]. Hypertension was considered as systolic BP ≥ 140 mmHg or systolic BP ≥ 90 mmHg or current use of antihypertensive medications [34].

Cardiovascular disease (CVD) was defined as any coronary heart disease (CHD) or stroke. Coronary heart disease was defined as myocardial infarction (MI), probable MI, unstable angina pectoris and angiographic proven CHD [35].

Atherogenic index of plasma (AIP; log TG/HDL) were calculated as cardiovascular risk factors parameter [36].

### 2.7. Statistical Methods

Dietary intake of l-arginine was adjusted for total energy intake, based on the residuals method [37]. Dietary total l-arginine was categorized into quartiles (<3.06, 3.06–3.92, 3.06–5.20, and ≥5.20 g/day). Mean (±SD) values and the proportions of participant characteristics across quartiles of dietary l-arginine intakes were compared using the analysis of variance or chi-square test, respectively. Serum NOx concentrations were compared across quartiles of dietary l-arginine intake using analysis of variance.

To evaluate the association of dietary l-arginine with serum NOx (β regression and 95% confidence interval), linear regression models with adjustment for potential confounders were used. A univariate analysis was performed for each potential confounder including age, medication usage, smoking, obesity, diabetes, hypertension, serum creatinine as well as the main nitrate–nitrite containing foods including high- and medium-nitrate containing vegetables, grains and processed meats; variables with *P_E_* < 0.2 in the univariate analysis were selected for the multivariable models; *P_E_* (*p* value for entry) determines which variables should be included in the final multivariable model. A linear trend test was performed by considering each ordinal score variable as a continuous variable in the model.

All statistical analysis were conducted using SPSS (Version 16.0; Chicago, IL, USA), and *p* < 0.05 were considered significant.

## 3. Results

Mean age of participants (39.2% men) was 45.9 ± 15.9 years. Mean dietary intakes of protein and l-arginine were 83.7 ± 42.5 g/day (82.5 ± 42.1 and 84.5 ± 42.8 g/day, in men and women, respectively) and 4.43 ± 2.56 g/day (4.31 ± 2.30 and 4.5 ± 2.72 g/day, in men and women, respectively), respectively. Lowest and highest categories of l-arginine intake, defined as the 10th and 90th percentiles, were <2.4 and ≥6.7 g/day, respectively. Mean dietary intake of l-arginine to total protein ratio was 0.053 ± 0.02. Dietary intakes of l-arginine were more from grains (33.0% ± 12.9%), meats (30.2% ± 12.4%), and dairy products (14.0% ± 7.5%); consumption of nuts and legumes had 4.0% ± 5.2% and 3.8% ± 5.0% contribution in total l-arginine intakes, respectively.

Characteristics of the study population across quartile categories of dietary arginine intakes are presented in Table 1. There was no significant difference in anthropometric and biochemical measures across the quartiles of l-arginine intakes. Dietary intakes of the study participants across quartiles of l-arginine intakes are shown in Table 2. There was no significant difference in total calorie, fat and carbohydrate intakes; dietary intakes of protein (16.1% *vs.* 11.4% total energy intakes, *p* < 0.01) and l-arginine to protein ratio (0.056 *vs.* 0.051, *p* < 0.01) were higher in the highest compared to the lowest quartile. Participants with higher intakes of l-arginine, also had higher intakes of main nitrate–nitrite containing foods including high- and medium-nitrate containing vegetables as well as grains and processed meats (*p* < 0.05).

**Figure 1:**
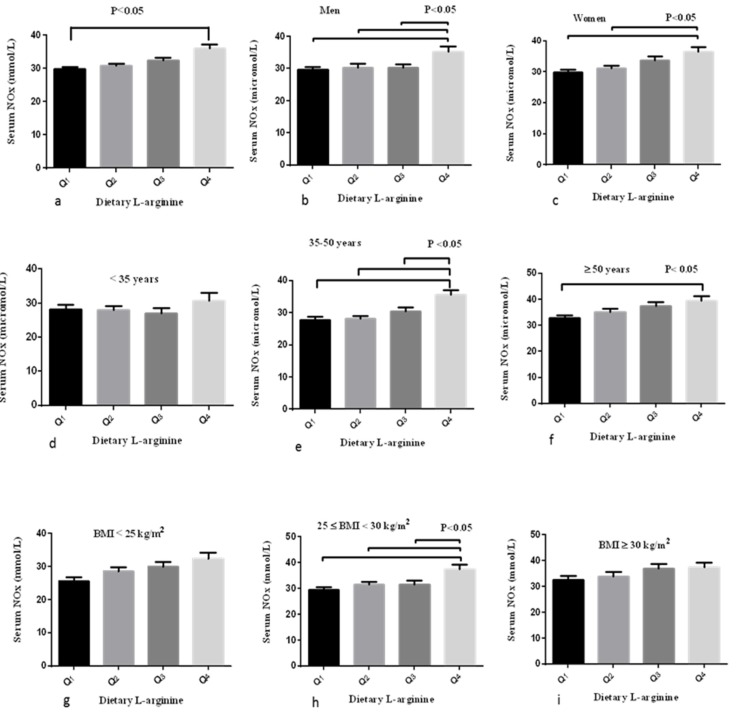
Mean serum NOx across quartile categories of l-arginine intakes stratified by sex, age and BMI categories. (**a**) Serum NOx was higher in the highest compared to the lowest quartile of l-arginine intakes (36.1 *vs.* 29.8 μmol/L, *p* < 0.05); (**b**) men who had higher l-arginine intake were significantly more likely to have higher levels of serum NOx (35.3 *vs.* 29.7 μmol/L, *p* < 0.05); (**c**) women who had higher l-arginine intake were significantly more likely to have higher levels of serum NOx (36.5 *vs.* 29.9 μmol/L, *p* < 0.05); (**d**) there was no significant difference in serum NOx across quartiles of l-arginine intakes in adults aged <35 years; (**e**) adults aged 35–50 years in the fourth quartile compared to other quartiles of l-arginine intakes had higher levels Serum NOx concentrations (*p* < 0.05); (**f**) higher serum NOx was observed in adults who consumed highest compared to the lowest quartile of dietary l-arginine (39.5 *vs.* 32.8 μmol/L, *p* < 0.05); (**g**) there was no significant difference in serum NOx across quartiles of l-arginine intakes in subjects with BMI < 25 m^2^/kg; (**h**) in subjects with BMI range 25–30 m^2^/kg, highest compared to other quartiles of l-arginine intakes was accompanied with higher serum NOx (37.5 *vs.* 29.6 μmol/L, *p* < 0.05); and (**i**) there was no significant difference in serum NOx across quartiles of l-arginine intakes in subjects with BMI ≥ 30 m^2^/kg. * *p* < 0.05 (analysis of variance and Bonferroni pairwise comparison test was used).

Mean serum NOx across quartile categories of l-arginine intakes are shown in Figure 1. Both men (35.3 *vs.* 29.7 μmol/L, *p* < 0.01) and women (36.5 *vs.* 29.9 μmol/L, *p* < 0.01) who had higher l-arginine intake were significantly more likely to have higher levels of serum NOx. Moreover, serum NOx concentrations increased across increasing intakes of l-arginine only in adults aged ≥35 years.

Associations (coefficient β and 95% CI) of dietary l-arginine intakes and serum NOx levels, stratified by sex, age- and BMI-categories, are presented in Table 3. After adjustment of all potential confounding variables, a significant positive association was observed between l-arginine intake and serum NOx concentrations in the third and fourth quartile of l-arginine (β = 5.3, 95% CI = 2.3, 8.3, and β = 10.6, 95% CI = 6.8, 14.4, *P* for trend = 0.001); this association was stronger in women in the upper quartile of l-arginine intakes. Further analysis stratified by age-categories showed that the positive association between l-arginine intake and serum NOx concentrations became stronger across increasing age (β = 3.1, 7.1, and 11.5 in young, middle-aged and older adults, respectively); the trend of these associations across quartiles of l-arginine was statistically significant. When the analysis was stratified by BMI categories, l-arginine intakes were strongly associated with serum NOx levels in overweight and obese subjects who were in the upper quartile (β = 7.6, 95% CI = 4.2, 11.0 and β = 8.1, 95% CI = 5.6, 14.0, in overweight and obese subjects, respectively); a borderline significant association was observed between l-arginine intake and serum NOx concentrations in young adults and normal weight subjects. When l-arginine was included in the models as continuous variable, similar findings were observed (Table 3).

Participants with higher intakes of l-arginine had also higher intakes of main nitrate–nitrite containing foods in their regular diet; considering that rich sources of nitrate-nitrite in the diet could affect serum NOx circulation, dietary intakes of high and medium NCVs as well as grains and processed meats were included as covariates in linear regression models. In fully adjusted linear regression model used to assess the relation between l-arginine intakes and serum NOx in the total population, coefficient β for high and medium NCVs, grains and processed meats were −0.038 (*p* = 0.05), −0.26 (*p* = 0.039), −0.10 (*p* = 0.23), and 0.017 (*p* = 0.58), respectively.

Further analysis stratified by HTN status showed a strong association between l-arginine intakes and serum NOx in non-HTN compared to HTN subjects (β = 2.65, 95% CI = 2.1–3.2 *vs.* β = 1.25, 95% CI = −1.64–4.15).

Linear association between dietary l-arginine intakes and serum NOx levels in non-hypertensive obese subjects was also greater than hypertensive obese subjects (β = 5.96, 95% CI = 0.17–11.7 *vs.* β = 1.09, 95% CI = −7.02–14.4).

Cardiovascular risk factors across categories of body mass index are presented in Table 4. There was an increasing trend in blood pressure, AIP, prevalent diabetes, HTN and CVD across increasing BMI (*p* for all < 0.05).

## 4. Discussion

Findings from this cross-sectional study indicated that higher dietary l-arginine intakes were strongly associated with serum NOx concentrations, a quantitative measure of NO production, independent of possible confounding variables, an association that should support previous reports regarding the promotional effect of l-arginine intake on systemic NO production. A stronger relation between l-arginine and NOx in women, older adults, overweight and obese subjects, as well as non-HTN subjects, as another important finding of our study, also indicated l-arginine/NO pathway may be affected by different physiological and pathophysiological conditions. The association between l-arginine intakes and serum NOx was independent of overall dietary patterns of the study participants (data not shown) and other dietary factors including high-nitrate containing foods, which may affect NO metabolism [38]. We also observed inverse associations between high- and medium-nitrate containing vegetables and serum NOx; we do not explain this finding from the results of this study. However, serum NOx has two sources, namely dietary and endogenous production [39,40]. In our study, NOx was measured in fasted subjects, when NOx is mostly derived from endogenous source. It could therefore be speculated that the presence of a diet that includes consuming high-nitrate containing foods, endogenous NOx production is lower.

In the current study, mean intake of dietary l-arginine (4.43 ± 2.56 g/day) was comparable with other populations, and l-arginine intakes were more from grains and meats and less from nuts and legumes. Recommended dietary allowance has not yet been defined for l-arginine intake; soy protein, peanuts, walnuts and fish meats are rich sources while cereals and grains contain lower levels of l-arginine. Different dietary patterns between populations, therefore, may account for differences in mean intakes of l-arginine. Mean arginine intake for the US adults is reported to be 4.40 g/day, with 25% of people consuming <2.6 g/day [2]. Median l-arginine intake in an adult population, participants of the National Health Nutrition and Examination Survey, was also estimated to be 3.8 g/day. The highest level (90th percentile) intake of l-arginine in our population (6.7 g/day) was also within the range of previous reports (4.5–7.5 g/day) [1].

An overview of previous studies investigating the effect of l-arginine intakes on NO synthesis indicates controversial findings; unlike a large number of experimental and clinical studies that directly confirm the improvement of NO-dependent endothelial function or l-arginine-NO production, there are relatively few studies that contradict these findings [13,34,35,36,37]. A significant dose-dependently increase was observed in NO synthesis following consumption of a diet containing high- compared to low-arginine (4.9 g *vs.* 1.8 g) [16]. Intravenous infusion of 3 g l-arginine in a cross-over design in type 2 diabetic and healthy subjects acutely increased serum NOx concentrations [41]. A substantial increase in NO production was also observed in atherosclerotic patients during oral supplementation of l-arginine in two doses of 6 and 12 g/day for 28 days [42]; after supplementation, serum NOx levels were unexpectedly higher in low-compared to high-dose of l-arginine (7.33 *vs.* 3.59 μM). In diabetic patients with atherosclerotic peripheral arterial disease, two-month oral supplementation with l-arginine 6 g/day) led to substantial increase in NO concentration [43]. In contrast, some investigations reported no major change in NO production following l-arginine supplementation; in a cross-over study of healthy individuals, plasma NOx was not affected by an l-arginine and NOx-free diet, compared to a NOx-free diet containing 3.8 g/day l-arginine [44]. Likewise, no significant change was observed in serum NOx concentrations between acute oral l-arginine supplementation at a dose of 6 g compared to placebo, 2 h after ingestion in healthy subjects; in this study, plasma levels of asymmetric dimethylarginine (ADMA), a competitive inhibitor of l-arginine metabolism by NO synthase, was similar between the groups at baseline [22].

The different response to l-arginine supplementation may be explained by different levels of endogenous ADMA; in subjects with low ADMA levels, l-arginine intakes had no significant effect while in subjects with high ADMA levels, l-arginine normalized l-arginine/ADMA ratio and promoted NO production [21,45].

Quantitative changes in NO production in response to various doses of l-arginine are less documented but in most clinical studies that reported a considerable effect of l-arginine on NO synthesis, its supplementation was over the usual dietary intake. In our study, the association of dietary l-arginine intake and serum levels of NOx was only significant in the upper intakes (median, 6.33 g/day). A significant trend in the association of dietary l-arginine and serum NOx, observed across quartiles of l-arginine intakes, may also consider as a dose-response effect; the trend of l-arginine-NOx association was only significant in overweight and obese compared to normal weight subjects. It has been proposed that dose-dependent relation between l-arginine intake and NO synthesis is dependent to basal NO synthesis status and it can be expected to be higher in pathophysiologic conditions with a lower total NO bioavailability [45]. Although there is no published data to support our speculation, another issue that seems to be important in the dietary l-arginine-NO production is dietary source of l-arginine. In our study, contribution pattern of food groups was different in the upper quartile of l-arginine intakes; in the highest, compared to the lowest quartile of l-arginine, its main sources was mainly from meats (39% *vs.* 24%) while grains were major source of l-arginine in the lowest quartile compared to the highest quartile (39% *vs.* 26%).

In our study, a positive association between l-arginine intake and serum NOx was only statistically significant in overweight and obese, but not in normal weight subjects. It is notable that the observed association in overweight and obese subjects was independent of potential confounding variables especially serum creatinine and HTN status of the participants. Further analysis stratified based on HTN status of obese patients showed that the positive association of l-arginine intakes and serum NOx was only significant in non-hypertensive obese subjects. We also showed that the association between dietary intakes of l-arginine and serum NOx levels, independent of BMI status, was impaired in hypertensive patients. Although overproduction of NO in overweight and obesity has mainly been attributed to increased inducible NOS (iNOS) activity in response to insulin and pro-inflammatory cytokines [28,46]. Considering the fact that an elevation of serum levels of ADMA occurs in an overweight status [47,48], a higher conversion of dietary l-arginine intake to NO synthesis is expected. Lack of association between dietary l-arginine intakes and serum NOx levels in hypertensive patients, in our study, may be explained by impaired l-arginine-NO pathway in HTN status, indicated by previous investigations [49,50].

The stronger association between l-arginine and serum NOx concentrations in middle-aged and older adults, compared to young adults, observed in our population, may also be related to higher levels of ADMA in aging [51,52]. In our study, l-arginine intake was statistically higher in women; however there was no significant difference in serum NOx (31.4 *vs.* 32.8 μmol/L, in men and women, respectively) between men and women.

The apparently simple association of l-arginine bioavailability and systemic NO synthesis is complicated by the “l-arginine paradox” [16,45] and it is beyond the capacity of the current study to clarify current challenges in this phenomenon. Main explanations for the “l-arginine paradox” include l-arginine-induced insulin secretion [53,54], reduced l-arginine production by endogenous NOS inhibitors, e.g., ADMA, l-citrulline, argininosuccinic acid, and agmatine [53,54], colocalization of arginine transporter (*i.e.*, cationic amino acid transporter-1) and eNOS in caveolae [54], and increased l-arginine-induced tetrahydrobiopterin (BH4) production [54,55]. l-arginine enhances guanosine-5′-triphosphate (GTP) cyclohydrolase I, the rate-limiting enzyme in *de novo* synthesis of BH4 [54]. On the other hand, extracellular l-arginine at physiological concentrations regulates iNOS activity and the higher levels of l-arginine lead to higher induction of iNOS [53].

The strengths of this study were a population-based setting, and use of a validated FFQ to assess regular dietary intake that provided an accurate estimation for dietary l-arginine intake.

Some limitations should be considered in interpretation of the findings. First, lack of data on serum levels of l-arginine was an important limitation of this study; however, an acceptable correlation has been reported between dietary l-arginine intakes and serum l-arginine in previous studies. Lack of data on ADMA, an important inhibitor of l-arginine-NO pathway, may also be considered a limitation. Due to the population-based nature of the current study, we were also unable to measure NOS activity to explain the l-arginine paradox as well as related plausible mechanisms that clarify the association between dietary l-arginine intakes and endogenous NO production.

The lack of separate measurements for nitrate and nitrite may be considered another limitation of our study; considering the higher concentration rate of nitrate, compared to nitrite, in serum samples, use of NOx may not accurately represent NO production rate [56]. It is not clear which, nitrite, nitrate or NOx, would useful for measurement of NO synthesis [27,57]. Nitrite has been considered as a more sensitive index because it is affected less by diet and kidney function [27,58]; on the other hand, it has been proposed that circulating nitrate/NOx would be a more reliable index of NO formation because nitrate is less prone to rapid changes caused by altered metabolism or destruction during sample preparation [27,59,60].

## 5. Conclusions

To our knowledge, this is the first attempt to elucidate the association between dietary l-arginine intakes and NO synthesis using a population-based study. In conclusion, our findings indicate a strong association between dietary intakes of l-arginine and serum NOx, as endogenous production of NO, an association that is affected by age, BMI and HTN status.

## Figures and Tables

**Table 1:** Characteristics of the study population across quartiles of dietary l-arginine intakes.

	Dietary l-Arginine				
	Q_1_ (*n* = 692)	Q_2_ (*n* = 693)	Q_3_ (*n* = 693)	Q_4_ (*n* = 693)	*p* *
l-arginine (g/day)					
Range	<3.06	3.06–3.92	3.92–5.20	≥5.20	
Median	2.55	3.49	4.46	6.33	
Age (years)	45.5 ± 16.2	46.1 ± 15.8	45.9 ± 15.5	46.0 ± 16.2	0.90
Men (%)	39.4	40.0	39.2	38.2	0.91
Smoking (%)	9.5	9.4	8.7	7.9	0.69
Body mass index (kg/m^2^)	27.6 ± 4.9	27.1 ± 4.8	27.7 ± 4.7	27.6 ± 4.7	0.13
Waist circumferences (cm)	91.8 ± 13.2	90.8 ± 12.9	92.1 ± 13.0	91.8 ± 12.9	0.27
Systolic blood pressure (mmHg)	116 ± 19.3	119 ± 17.2	117 ± 18.2	117 ± 19.4	0.56
Diastolic blood pressure (mmHg)	72.5 ± 10.3	72.9 ± 10.5	73.6 ± 10.2	72.7 ± 10.7	0.24
Fasting blood glucose (mg/dL)	97.7 ± 31.3	98.0 ± 30.1	97.2 ± 30.4	98.9 ± 33.8	0.79
Total cholesterol (mg/dL)	191 ± 38.0	193 ± 42.3	194 ± 34.0	193 ± 41.0	0.49
Triglycerides (mg/dL)	153 ± 104	158 ± 106	156 ± 92.0	149 ± 93.0	0.41
HDL-C (mg/dL)	42.8 ± 10.6	42.5 ± 10.2	42.3 ± 9.7	43.3 ± 10.1	0.29
LDL-C (mg/dL)	119 ± 33.0	119 ± 32.8	121 ± 33.1	119 ± 32.3	0.46
Serum creatinine (μmol/L)	93.0 ± 18.6	91.4 ± 16.8	91.5 ± 16.1	91.0 ± 15.0	0.15
Hypertension (%)	16.0	18.1	14.3	18.0	0.19
History of cardiovascular disease (%)	2.9	3.9	4.1	4.7	0.40

Data are mean ± SD (unless stated otherwise); * analysis of variance for continuous variables and chi square test for dichotomous variables were used.

**Table 2:** Dietary intakes of the participants across quartiles of l-arginine intakes.

	Dietary l-Arginine			
	Q_1_ (*n* = 692)	Q_2_ (*n* = 693)	Q_3_ (*n* = 693)	Q_4_ (*n* = 693)
Dietary l-arginine (g/day)				
Range	<3.06	3.06–3.92	3.92–5.20	≥5.20
Median	2.55	3.49	4.46	6.33
Energy intake (kcal/day)	296151.0	2178 ± 32.0	2099 ± 29.8	2638 ± 48.6
Carbohydrate (% energy)	55.8 ± 0.3	57.9 ± 0.3	58.8 ± 0.2	56.5 ± 0.3
Protein (% energy)	11.4 ± 0.1	12.8 ± 0.1	14.1 ± 0.1	16.1 ± 0.1
Total fats (% energy)	35.1 ± 0.3	31.7 ± 0.2	29.7 ± 0.2	30.0 ± 0.3 *
Total vegetables (g/day)	275 ± 7.1	270 ± 6.9	305 ± 6.9	342 ± 6.9 *
High-nitrate vegetables (g/day)	35.4 ± 1.4	25.1 ± 1.5	42.0 ± 1.5	43.4 ± 1.5 *
Medium-nitrate vegetables (g/day)	31.1 ± 1.3	31.2 ± 1.3	33.5 ± 1.3	41.3 ± 1.3 *
Low-nitrate vegetables (g/day)	280 ± 9.0	273 ± 9.1	275 ± 8.8	262 ± 9.2 *
Grains (g/day)	15.5 ± 1.9	20.4 ± 1.9	23.4 ± 1.9	33.9 ± 1.9 *
Processed meats	7.2 ± 0.6	7.9 ± 0.5	8.1 ± 0.5	11.8 ± 0.5 *

Data are Data are mean ± SE; analysis of covariance was used with adjustment for total energy intakes; * *p* < 0.05.

**Table 3:** The association of dietary l-arginine intakes and serum NOx according to sex and age groups.

	Dietary l-Arginine (mg/Day)				
	l-Arginine as Continuous	Q_2_ (3.06–3.92)	Q_3_ (3.92–5.20)	Q_4_ (≥5.20)	*p* for Trend ^3^
Total	0.96 (0.63, 1.29) ^1^ 1.67 (1.03, 2.32) ^2^	1.07 (−1.28, 3.43) ^1^ 1.24 (−1.24, 3.73) ^2^	2.63 (0.27, 4.99) ^1^ 2.89 (−0.42, 5.37) ^2^	6.28 (3.92, 8.64) ^1^ 6.63 (4.14, 9.12) ^2^	0.003 0.001
Men (*n* = 1086)	0.22 (−0.31, 0.74) ^1^ 0.35 (−0.54, 1.24) ^2^	0.78 (−2.61, 4.18) ^1^ 1.22 (−2.48, 4.92) ^2^	0.69 (−2.72, 4.11) ^1^ 1.61 (−2.19, 5.40) ^2^	5.64 (2.20, 9.08) ^1^ 4.36 (0.57−8.14) ^2^	0.121 0.024
Women (*n* = 1685)	1.29 (0.87, 1.71) ^1^ 2.69 (1.78, 3.61) ^2^	1.27 (−1.93, 4.48) ^1^ 0.93 (−2.72, 4.57) ^2^	3.89 (0.68, 7.09) ^1^ 3.49 (−0.16, 7.16) ^2^	6.67 (3.49, 9.86) ^1^ 6.12 (6.52, 9.72) ^2^	0.016 0.001
Age-categories					
<35 years (*n* = 788)	1.50 (0.95, 2.04) ^1^ 0.17 (−0.46, 0.81) ^2^	0.11 (−3.75, 3.97) ^1^ 0.55 (−3.87, 4.33) ^2^	0.91 (−3.01, 4.83) ^1^ 1.74 (−2.31, 6.32) ^2^	3.68 (−0.13, 7.50) ^1^ 6.07 (0.44, 11.7) ^2^	0.327 0.051
35–50 years (*n* = 911)	0.77 (0.19, 1.35) ^1^ 0.58 (−0.10, 1.27)^2^	0.29 (−3.69, 4.27) ^1^ 0.73 (−3.90, 5.67) ^2^	1.91 (−1.98, 5.80) ^1^ 2.27 (−2.96, 6.38) ^2^	8.27 (4.26, 12.2) ^1^ 9.12 (3.99, 13.61) ^2^	0.003 0.001
≥50 years (*n* = 1072)	0.73 (0.20, 1.27) ^1^ 0.91 (0.30, 1.52) ^2^	2.28 (−1.83, 6.41) ^1^ 3.39 (−0.89, 7.69) ^2^	4.54 (0.37, 8.71) ^1^ 7.07 (2.46, 11.6) ^2^	6.62 (2.48, 10.7) ^1^ 12.1 (6.48, 17.7) ^2^	0.167 0.050
BMI-categories					
<25 kg/m^2^ (*n* = 808)	0.12 (−0.46, 0.70) ^1^ 0.36 (−1.02, 1.70) ^2^	1.07 (−2.65, 4.79) ^1^ 0.98 (−2.87, 4.83) ^2^	2.49 (−1.37, 6.36) ^1^ 3.57 (−0.66, 7.81) ^2^	4.87 (1.02, 8.72) ^1^ 8.12 (−2.83, 13.4) ^2^	0.888 0.091
25–30 kg/m^2^ (*n* = 1147)	0.26 (−0.46, 0.99) ^1^ 1.02 (−0.83, 2.87) ^2^	1.84 (−1.95, 5.65) ^1^ 2.38 (−1.57, 6.33) ^2^	2.09 (−1.76, 5.94) ^1^ 3.36 (−0.82, 7.54) ^2^	7.85 (3.98, 11.7) ^1^ 10.7 (5.43, 16.0) ^2^	0.160 0.024
≥30 kg/m^2^ (*n* = 816)	1.06 (0.57, 1.55) ^1^ 2.12 (1.30, 2.94) ^2^	1.17 (−3.83, 6.17) ^1^ 2.78 (−2.34, 7.91) ^2^	4.29 (−0.36, 8.95) ^1^ 7.39 (2.05, 12.7) ^2^	4.85 (0.21, 9.49) ^1^ 11.0 (4.29, 17.5) ^2^	0.027 0.001

*Q_1_* (<3.06 mg/day) was considered as a reference group; data are β regression and 95% confidence interval; ^1^ unadjusted model; ^2^ multiple regression models with adjustment for sex, age (continues), obesity (yes/no), smoking (yes or no), serum creatinine (μmol/L), diabetes (yes/no), hypertension (yes/no), medications (yes/no) and daily energy intake (kcal/day); ^3^ a linear trend test was performed by considering each ordinal score variable as a continuous variable in the model; median of dietary l-arginine was 2.55, 3.49, 4.46, and 6.33 g/day in the first, second, third and fourth quartile category, respectively.

**Table 4:** Cardiovascular risk factors across categories of body mass index.

	Body Mass Index (kg/m^2^)		
	<25 (*n* = 808)	25–30 (*n* = 1147)	≥30 (*n* = 816)
Systolic blood pressure (mmHg)	110 ± 18.2	116 ± 18.0	124 ± 18.8 *
Diastolic blood pressure (mmHg)	68.9 ± 9.6	73.3 ± 9.7	77.4 ± 10.2 *
Serum creatinine (μmol/L)	91.7 ± 16.5	91.4 ± 16.5	92.5 ± 17.5
Atherogenic index of plasma	0.37 ± 0.27	0.53 ± 0.29	0.61 ± 0.27 *
Diabetes (%)	8.5	11.2	19.0 *
Hypertension (%)	8.9	15.0	28.8 *
History of cardiovascular disease (%)	2.5	4.3	4.8 *

Data are mean ± SD (unless stated otherwise); analysis of variance or chi square test was used; * *p* < 0.05 (significant difference with BMI < 25 kg/m^2^).
